# Sources of HIV incidence among stable couples in sub-Saharan Africa

**DOI:** 10.7448/IAS.17.1.18765

**Published:** 2014-02-21

**Authors:** Hiam Chemaitelly, Susanne F Awad, James D Shelton, Laith J Abu-Raddad

**Affiliations:** 1 Infectious Disease Epidemiology Group, Weill Cornell Medical College in Qatar, Cornell University, Qatar Foundation, Education City, Doha, Qatar; 2 Bureau for Global Health, United States Agency for International Development, Washington, DC, USA; 3 Department of Healthcare Policy and Research, Weill Cornell Medical College, Cornell University, New York, USA; 4 Vaccine and Infectious Disease Division, Fred Hutchinson Cancer Research Center, Seattle, Washington, DC, USA

**Keywords:** stable couples, sources of infection, HIV incidence, Sub-Saharan Africa, demographic and health surveys, mathematical model

## Abstract

**Introduction:**

The recent availability of efficacious prevention interventions among stable couples offers new opportunities for reducing HIV incidence in sub-Saharan Africa. Understanding the dynamics of HIV incidence among stable couples is critical to inform HIV prevention strategy across sub-Saharan Africa.

**Methods:**

We quantified the sources of HIV incidence arising among stable couples in sub-Saharan Africa using a cohort-type mathematical model parameterized by nationally representative data. Uncertainty and sensitivity analyses were incorporated.

**Results:**

HIV incidence arising among stable concordant HIV-negative couples contribute each year, on average, 29.4% of total HIV incidence; of those, 22.5% (range: 11.1%–39.8%) are infections acquired by one of the partners from sources external to the couple, less than 1% are infections acquired by both partners from external sources within a year and 6.8% (range: 3.6%–11.6%) are transmissions to the uninfected partner in the couple in less than a year after the other partner acquired the infection from an external source. The mean contribution of stable HIV sero-discordant couples to total HIV incidence is 30.4%, with most of those, 29.7% (range: 9.1%–47.9%), being due to HIV transmissions from the infected to the uninfected partner within the couple. The remaining incidence, 40.2% (range: 23.7%–64.6%), occurs among persons not in stable couples.

**Conclusions:**

Close to two-thirds of total HIV incidence in sub-Saharan Africa occur among stable couples; however, only half of this incidence is attributed to HIV transmissions from the infected to the uninfected partner in the couple. The remaining incidence is acquired through extra-partner sex. Substantial reductions in HIV incidence can be achieved only through a prevention approach that targets all modes of HIV exposure among stable couples and among individuals not in stable couples.

## Introduction

The majority of the adult population in sub-Saharan Africa (SSA) live in marital or co-habiting couples [1]. A considerable fraction of these stable couples (SCs) are HIV sero-discordant (that is, one partner testing HIV sero-positive while the other testing HIV sero-negative) [1–5]. Recent randomized clinical trials have demonstrated the potential for averting much of the HIV heterosexual transmission among stable HIV sero-discordant couples (SDCs) using highly efficacious HIV prevention interventions such as antiretroviral therapy (ART) [6–9]. This progress in HIV prevention research revealed new horizons for HIV policy and programming in SSA and placed SDCs on the list of priorities for HIV prevention efforts [10–12]. Yet, reaching a consensus on an effective HIV prevention strategy in SSA that factors SDCs entails a comprehensive understanding of the sources of HIV incidence arising in the population, particularly among SCs.

We recently quantified the contribution of HIV incidence within SDCs to total HIV population-level incidence (SDC_*int*_) across SSA [13]. We also estimated the fraction of HIV infections arising among SDCs that are due to sources external to the couple [14]. We further quantified the risk of HIV transmission from the infected to the uninfected partner in the SDC [15]. In this article, we complement our work by extending our analysis to the sources of HIV incidence across the different types of SCs for 24 countries in SSA. Specifically, we assess the contribution of HIV incidence among stable concordant HIV-negative couples (SCNCs) relative to total HIV population-level incidence where: a) one of the partners acquires the infection from a source external to the couple (SCNC_*ext*×1_), b) each of the partners acquires the infection from a source external to the couple (SCNC_*ext*×2_) and c) one partner acquires the infection from a source external to the couple and then transmits the infection to the uninfected partner in the couple shortly after acquiring the infection (SCNC_*ext*+int_). We also estimate the contribution of HIV incidence among SDCs where the uninfected partner acquires the infection from a source external to the couple (SDC_*ext*_). We further provide an estimate for the incidence contribution of individuals not in a SC where HIV infection is acquired through sexual contacts outside of the context of marital or cohabiting partnerships (NSC). Finally, we update the results of our earlier work on SDCs [13, 14] through the use of recently available survey data, in addition to including countries that were not covered earlier, and the use of a more elaborate mathematical modeling framework for generating these estimates.

## Methods

We constructed a cohort-type mathematical model that estimates the contribution of HIV incidence stratified by couple status and source of infection to total HIV incidence in the population over the course of a year. The model uses the calculated risks of HIV transmission and acquisition among SCs to derive the annual number of new HIV infections arising among SCs and the relative contribution of each source of incidence to the total HIV incidence in the population.

### Model structure and measures’ definition

**Figure 1:**
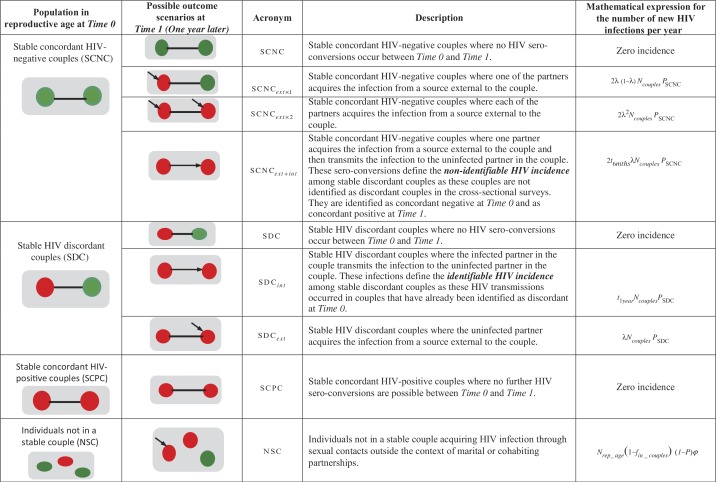
**Model conceptualization for HIV incidence in the population classified based on the sero-status of stable couples and source of infection. The table shows the possible outcome scenarios and the associated mathematical expressions for the different HIV incidence measures. The green circle indicates an HIV sero-negative individual, while the red circle indicates an HIV sero-positive individual**. *Parameters include *λ*: the probability of an HIV sero-negative partner in a stable couple (SC) to acquire the infection from a source external to the couple over the course of one year; *N*_*couples*_: the number of SCs identified in the baseline screening cross-sectional survey at *Time 0*; *P*_SCNC_: the prevalence of stable concordant HIV-negative couples among all couples; *P*_SDC_: the prevalence of stable HIV discordant couples among all couples; *t*_6*mths*_: the probability that the index partner who acquired the infection from an external source will transmit the infection to the uninfected partner during the six months following the acquisition of HIV; *t*_1*year*_: the probability that the index partner in a stable HIV discordant couple will transmit the infection to the uninfected partner during the time between the two cross-sectional surveys at *Time 0* and *Time 1*; *N*_*rep_age*_: the size of the population in reproductive age; *f*_*in_couples*_: the fraction of the population in reproductive age engaged in SCs; *P*: HIV prevalence in the population; *ϕ*: the HIV population-level incidence rate.

Our model sets out to ask the following question: if there is a national screening survey at a point in time (at *Time 0*) that identifies individuals engaged in SCs and others not engaged in SCs, what proportion of these individuals would acquire HIV over the following year (at *Time 1*), that is within a year of observation, and how? Figure 1 shows the possible changes to the HIV sero-status of the three types of SCs that are identified in the baseline cross-sectional survey at *Time 0* (SCNCs, SDCs or concordant HIV-positive couples), and changes to the HIV sero-status of individuals not in SCs, one year later at *Time 1*. The mathematical expressions used to assess the annual number of new HIV infections arising among these population sub-groups are also summarized in this figure and discussed further in the Supplementary file.

In summary, we drew a map of HIV incidence stratified by couple status and source of infection by measuring, for each country, the contribution of six different types of HIV incidence to total HIV incidence arising in the population over the course of a given year of observation (Figure 1). The latter is defined as the number of new HIV infections arising within a year among susceptible individuals in reproductive age. This measure defines the denominator in all six contribution measures, and is calculated using HIV population-level incidence rate and the estimated number of uninfected individuals in reproductive age for each country (Table 1 and Supplementary file).

**Table 1:** Key demographic and HIV-related indicators across the 24 sub-Saharan African countries included in our analysis

Country	Year	Pop in rep age	Fraction of pop in rep age that is in stable couples (%)	Number of stable couples	HIV pop prev (%)	Couples tested	Prev of stable discordant couples (%)	Prev of stable concordant positive couples (%)	Prev of stable concordant negative couples (%)	Fraction of HIV infected females in SDCs (%)	Fraction of circumcised males in SDCs with HIV infected females (%)	MC in the pop (%)	Condom use at last sex among couples (%)	HIV pop inc	HIV pop inc rate¥	λ^¢^
Senegal	2011	11,248,786	54.2	3,045,609	0.5	1586	0.9	0.4	98.8	35.8	100*	98.3*	2.0	8954	0.08	0.03
Niger	2006	4,714,950	76.3	1,798,871	0.7	2035	1.0	0.2	98.9	38.9	91.1	99.4	0.2	4683	0.10	0.04
Burkina Faso	2010	14,978,556	71.5	5,355,583	1.0	4894	1.2	0.2	98.6	56.8	90.9	88.4	3.6	10,378	0.07	0.02
Mali	2006	5,097,581	74.9	1,909,554	1.2	2467	1.2	0.4	98.5	72.1	94.2	97.7	0.7	7051	0.14	0.07
Congo	2007	29,807,400	61.4	9,147,146	1.3	2145	1.6	0.2	98.2	64.8	100	97.5	1.9	35,315	0.12	0.05
Burundi	2010	7,783,616	58.6	2,282,156	1.4	1933	1.1	1.1	97.8	59.8	40.2	30.9	1.6	9974	0.13	0.06
Ethiopia	2011	73,908,450	59.8	22,100,474	1.4	6183	1.1	0.6	98.4	60.4	93.0	92.4	0.5	94,707	0.13	0.07
Sierra Leone	2008	1,908,630	69.1	659,670	1.5	1576	1.7	0.5	97.8	58.8	100	97.9	1.0	2821	0.15	0.06
Liberia	2007	1,519,713	60.4	459,143	1.5	2255	1.9	0.3	97.9	61.6	100	98.7	2.5	2036	0.14	0.05
Guinea	2005	3,822,104	69.2	1,321,492	1.6	1851	1.6	0.4	98.1	41.0	93.6	99.0	0.8	5370	0.14	0.06
Ghana	2003	8,523,900	57.8	2,464,046	2.0	1811	2.7	1.0	96.3	45.6	100	95.2	3.4	16,700	0.20	0.07
Rwanda	2010	9,864,384	51.2	2,525,282	3.1	2808	2.2	2.4	95.4	40.6	28.5	13.3	5.1	16,251	0.17	0.04
Congo-Brazzaville	2009	335,136	55.4	92,749	3.3	2427	4.7	1.0	94.3	59.5	99.2	99.2	9.4	1006	0.31	0.11
Cameroon	2011	17,766,494	56.9	5,052,791	4.3	2845	5.9	1.5	92.6	52.9	96.0	94.1	6.7	61,241	0.36	0.11
Cote d'Ivoire	2005	9,218,355	51.7	2,383,175	4.7	1266	5.6	1.3	93.1	62.7	100	96.6	4.6	37,612	0.43	0.17
Uganda	2011	31,770,463	61.3	9,742,412	5.2	4774	6.3	3.4	90.3	48.1	37.6	26.7	3.9	252,995	0.84	0.40
Tanzania	2007	15,983,193	58.6	4,678,680	5.7	2810	6.4	2.4	91.2	45.7	55.0	67.1	4.9	88,897	0.59	0.22
Kenya	2008	17,986,100	54.9	4,933,587	6.4	1228	6.0	3.1	91.0	54.1	79.2	86.0	3.4	90,948	0.54	0.22
Malawi	2010	5,505,484	63.1	1,737,393	10.7	3340	8.4	6.2	85.4	45.0	35.0	21.6	5.5	27,049	0.55	0.10
Mozambique	2009	19,920,615	69.6	6,935,362	11.5	2494	9.7	4.5	85.8	50.7	37.7	51.8	3.2	211,557	1.20	0.51
Zambia	2007	4,276,800	58.9	1,254,813	14.2	2300	11.0	7.8	81.1	40.3	10.6	12.9	6.6	42,561	1.16	0.38
Zimbabwe	2011	11,213,332	57.3	3,209,816	15.3	2368	11.2	10.2	78.6	40.1	15.0	9.2	8.3	99,702	1.05	0.30
Swaziland	2006	525,600	35.3	92,847	18.9	659	16.4	28.8	54.8	53.0	17.8	8.2	23.9	14,068	3.30	2.47
Lesotho	2009	962,189	47.9	230,420	23.0	805	17.2	18.7	64.0	44.4	62.3	52.0	24.1	19,789	2.67	1.55

Countries are shown in order of increasing HIV prevalence in the population. Pop: population; Rep: reproductive; Prev: prevalence; SDC: stable HIV discordant couples; MC: male circumcision; Inc: incidence that is the number of new HIV infections per year.

* Data on male circumcision were not collected during the 2010–2011 round of the DHS for Senegal. The rates used are drawn from a previous Senegal DHS survey conducted in 2005

¥ HIV population-level incidence rate estimated by SPECTRUM or derived using DHS HIV prevalence per 100 person-years

λ^¢^ mean probability of acquiring HIV from sources external to the couple per 100 person-years derived by performing 10,000 runs of model fits.

Contributions among SCNCs:

1. SCNC_*ext*×1_ is the contribution of new HIV infections arising among SCNCs where only one of the partners acquires the infection from a source external to the couple, relative to total HIV incidence in the population. The number of infections here is calculated using the probability of acquiring the infection from a source external to the couple by one of the partners and the number of SCNCs in the population (Figure 1 and Supplementary file).
2. SCNC_*ext*×2_ is the contribution of new HIV infections arising among SCNCs where both partners acquire the infection from a source external to the couple within the same year, relative to total HIV incidence in the population in that year. The number of infections here is calculated using the probability of acquiring the infection from a source external to the couple by both partners and the number of SCNCs in the population (Figure 1 and Supplementary file).
3. SCNC_*ext*+*int*_ is the contribution of new HIV infections arising among SCNCs where the index partner acquires the infection from a source external to the couple, on average half the way through the year of observation, and transmits HIV to the other partner during the following half of the year, relative to total HIV incidence in the population. The number of infections here is calculated using the probability of acquiring the infection from a source external to the couple by one of the partners, the likelihood of transmitting the infection to the uninfected partner within six months following HIV acquisition and the number of SCNCs in the population (Figure 1 and Supplementary file).

Contributions among SDCs:

1. SDC_*int*_ is the contribution of new HIV infections arising among SDCs where the HIV infected partner transmits the infection to the uninfected partner, relative to total HIV incidence in the population. The number of infections here is calculated using HIV transmission probability per partnership over the course of a year of observation and the number of SDCs in the population (Figure 1 and Supplementary file).
2. SDC_*ext*_ is the contribution of new HIV infections arising among SDCs where the uninfected partner acquires HIV from a source external to the couple, relative to total HIV incidence in the population. The number of infections here is calculated using the probability of acquiring the infection from a source external to the couple and the number of SDCs in the population (Figure 1 and Supplementary file).

Contribution among individuals not in SCs:

1. NSC is the contribution of new HIV infections arising among susceptible individuals in reproductive age who are not part of a SC, relative to total HIV incidence in the population. The number of infections here is calculated using HIV population-level incidence rate and the number of susceptible individuals who are in reproductive age but not part of a SC (Figure 1 and Supplementary file).

### Model parameterization

Multiple data sources were used to obtain the model parameters with the primary source being the Demographic and Health Surveys (DHS), which are standardized nationally representative household-based surveys [16]. We analyzed the most recent DHS data for 24 countries in SSA where an HIV serological biomarker survey has been conducted [16]. These data were complemented by population size information from the United Nations World Population Prospects Database [17] to calculate country-specific demographic, behavioural and epidemiological indicators (Table 1).

As per DHS, a SC is defined as a man and a woman living in a consensual union within a household at the time of the DHS cross-sectional survey [18]. Accordingly, polygamous arrangements may contribute multiple SCs. SCs where one or both partners did not test for HIV were excluded from our analysis. Missing HIV information among all SCs ranged from 0.5% to 27.3% (mean of 10.8%) across countries. DHS guidelines were followed in applying to our calculations the sampling weights retrieved from the DHS datasets [18, 19]. Further details related to the management of DHS datasets can be found in the Supplementary file.

Following the methodology applied in our earlier work [13], we calculated the total HIV incidence in the population using HIV population-level incidence rates estimated using the Joint United Nations Programme on HIV/AIDS (UNAIDS) SPECTRUM model [20]. When these were not available, HIV population-level incidence rates were derived from the DHS HIV prevalence measures using the expression HIV $\text{HIV population-level incidence rate}=\frac{\text{HIV population prevalence}}{\text{Duration of infection}}$ [21]. Further details can be found in the Supplementary file.

To estimate HIV incidence arising among susceptible individuals that are not in SCs, we assumed that the risk of HIV acquisition among these individuals is equal to the average HIV population-level incidence rate among all susceptible individuals in the population (Figure 1 and Supplementary file) [14]. Country-specific values of the HIV population-level incidence rates can be found in Table 1.

The risk of HIV transmission within SDCs was calculated using the best available empirical evidence for HIV transmission probability per heterosexual coital act (*p*) as measured in the Rakai Study [22] and in the Partners in Prevention HSV/HIV Transmission Study (Partners in Prevention Study) (Table 2) [23, 24]. To adjust for male circumcision (MC) and self-reported condom use at last sex among SCs, we varied the efficiency of HIV transmission among SDCs reporting one or both interventions by applying to *p* multiplicative factors adjusting for the efficacy of MC and condom use in preventing HIV transmission. The effect of MC was stratified based on whether the index partner in the couple is a male or a female. We incorporated the coverage of condom use through a fraction of the acts that are protected by the partial efficacy of this intervention. The mathematical expressions and further details on the calculation of the risk of HIV transmission in presence of these interventions can be found in the Supplementary file.

**Table 2:** Model assumptions in terms of key parameter values related to HIV transmission and acquisition in sub-Saharan Africa

Assumptions	Parameter values	Source
Probability of acquiring HIV from sources external to the couple per 100 person-years (*λ*)	Derived from model fits	Derived
Probability of acquiring HIV by an individual not in a stable couple per 100 person-years (*ϕ*)	HIV-population-level incidence rate estimated by SPECTRUM or derived using DHS HIV prevalence	[16, 20]
HIV transmission probability per coital act (*p*)		
Acute infection (*p*_*acute*_)	0.036	[22, 25]
Latent infection (*p*_*latent*_)	0.0008	[22]
Average (*p*) using the Rakai and the Partners in Prevention Studies	0.00115	Derived
Average (*p*) using the Rakai Study	0.0012	[22]
Average (*p*) using the Partners in Prevention Study	0.0011	[23, 24, 26]
Frequency of coital acts per month (*n*)	8.3 acts per month	[22]
Demographic and epidemiological measures		
Number of individuals in reproductive age in the population (*N*_*rep_age*_)	Table 1	[16]
Number of stable sexual couples identified in baseline screening survey (*N*_*couples*_)	Table 1	[16]
Fraction of the population in reproductive age that are engaged in stable couples (*f*_*in_couples*_)	Table 1	[16]
HIV prevalence in the population (*P*)	Table 1	[16]
Prevalence of stable concordant HIV-negative couples (*P*_SCNC_)	Table 1	[16]
Prevalence of stable HIV discordant couples (*P*_SDC_)	Table 1	[16]
Fraction of females (index partners) among those initially concordant HIV-negative couples (*f*_*index*SCNC_)	50%	Assumption based on Eyawo et al. [27]
Fraction of females (index partners) among HIV discordant couples (*f*_*index*SDC_)	Table 1	[16]
Fraction of circumcised males among concordant HIV-negative couples (*f*_*mcpop*_)	Equal to fraction of circumcised males in the population (Table 1)	[16]
Fraction of circumcised males among HIV discordant couples where the female is HIV infected (*f*_*mcindex*_)	Table 1	[16]
Fraction of coital acts protected by condom use (*f*_*condom*_)	Table 1	[16]
Efficacy of condoms in preventing HIV transmission per condom-protected coital act (*E*_*condom*_)	80%	[24, 28]
Efficacy of male circumcision in preventing HIV acquisition among males per coital act (*E*_*mc*_)	58%	[29–32]
Duration		
Between each round of the cross-sectional survey (*τ*_*follow-up*_)	1 year	Convention for this model
Acute infection (*τ*_*acute*_)	49 days	[25]
Latent infection spent by index partner between two subsequent cross-sectional surveys (*τ*_*latent*_)	134 days	Derived

The likelihood of an HIV sero-negative partner in a SC acquiring the infection from a source external to the SC (defined here as *λ*) was determined by the condition that the total HIV incidence in the population, as estimated from pooling together all incidence measures among SCs and individuals not in SCs, must be equal to the total HIV incidence as estimated independently using the SPECTRUM model or derived using the DHS HIV prevalence (Table 1). All incidence measures were then recalculated using this fitted value of *λ*, and estimates for the contributions of new HIV infections among SCs to total HIV incidence in the population were derived (Figure 1 and Supplementary file).

### Uncertainty and sensitivity analyses

For each country, uncertainty analyses were performed for the estimates of the contribution of each type of HIV incidence by implementing 10,000 runs of the model using Monte Carlo sampling from triangular probability distributions for the ranges of demographic, biological and epidemiological parameters (Table S1 in the Supplementary file). Parameter ranges were primarily determined by the 95% confidence intervals (CIs) around the country-specific DHS measures. In the event where CIs were not available to provide a range, plausibility ranges informed by the range of available data in the literature or general consensus in the field were used as parameter ranges. In each run, model fits were conducted and an estimate for the probability of acquiring HIV from sources external to the couple (*λ*) was derived. Country-specific distributions for the estimated contributions of SCs to total HIV incidence by couple status and source of infection were then generated and used to calculate the mean and associated 95% CIs of these estimates (Figures 3 and 4).

**Figure 2:**
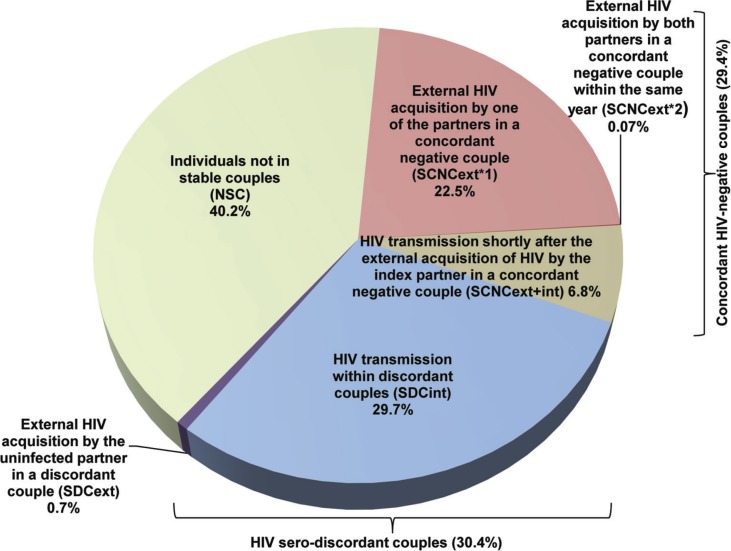
The average contributions to the total number of new HIV incident infections in a year in the population stratified by couples’ sero-status and source of HIV infection for 24 countries in sub-Saharan Africa. The average for each mode of exposure represents an average over the country-specific mean contribution measures (fraction of new HIV infections relative to total HIV incidence in the population in a given year). For each country, the mean contribution of each source of exposure to total HIV incidence was calculated based on 10,000 runs of the model using Monte Carlo sampling from triangular probability distributions for the specified ranges of model parameters.

Sensitivity analyses were conducted, using Kenya as an example, to assess the sensitivity of the calculated contribution measures to variations in level of condom use at last sex among SCs, MC coverage in the population and the fraction of the population in reproductive age that are engaged in SCs (Figure S2 in the Supplementary file).

## Results

**Figure 3:**
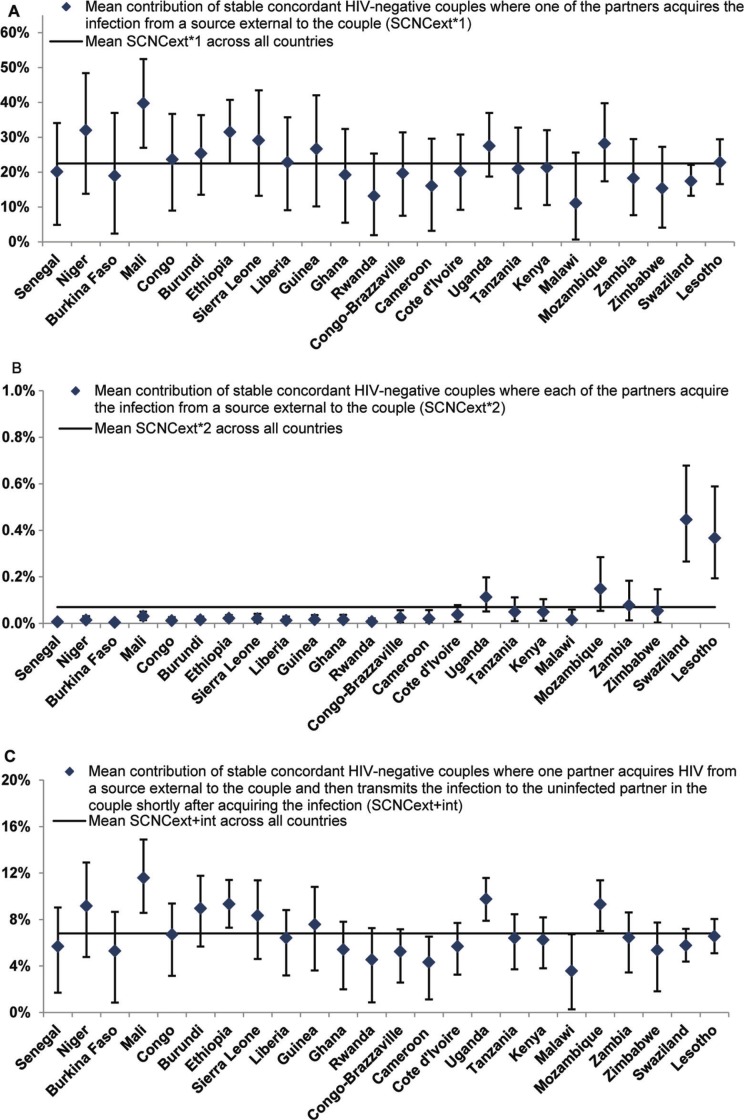
Mean and 95% confidence interval of the contributions of HIV incidence among stable concordant HIV-negative couples to total HIV incidence in the population in 24 countries in sub-Saharan Africa. The figure shows the contribution of HIV incidence among stable concordant HIV-negative couple where: (A) one partner acquires the infection from a source external to the couple, (B) each of the partners acquire the infection from a source external to the couple and (C) one partner acquires the infection from a source external to the couple and then transmits it to the uninfected partner in the couple. Estimates were calculated based on 10,000 runs of the model for each country using Monte Carlo sampling from triangular probability distributions for the specified ranges of uncertainty of the model parameters. Countries are shown in order of increasing HIV prevalence. The horizontal line in the different panels represents the average for the contribution measure in question across all countries.

**Figure 4:**
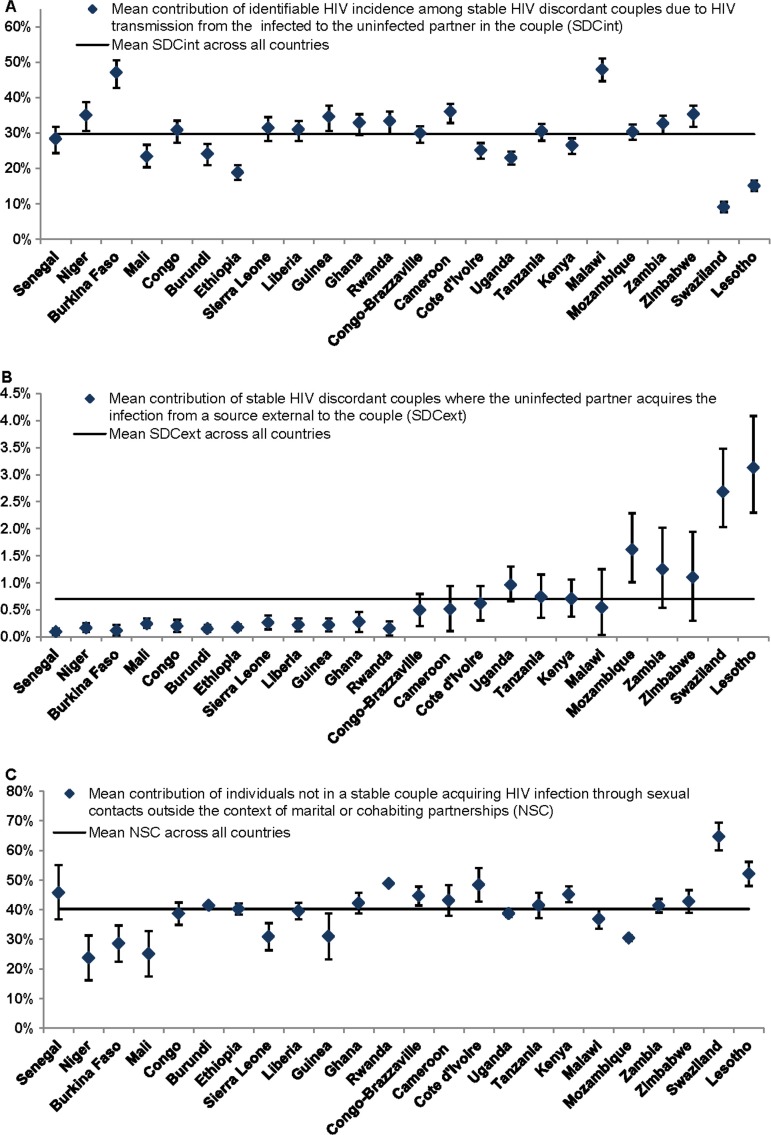
Mean and 95% confidence interval of the contributions of: (A) identifiable HIV incidence among stable HIV discordant couples due to HIV transmission from the infected to the uninfected partner in the couple, (B) HIV incidence among stable HIV discordant couples due to acquiring the infection from a source external to the couple and (C) HIV incidence among individuals not in stable couples. These measures, for 24 countries in sub-Saharan Africa, are relative to total HIV incidence in the population in each country. Estimates were calculated based on 10,000 runs of the model for each country using Monte Carlo sampling from triangular probability distributions for the specified ranges of uncertainty of the model parameters. Countries are shown in order of increasing HIV prevalence. The horizontal line in the different panels represents the average for the contribution measure in question across all countries.

The key demographic and HIV-related indicators for the 24 countries in SSA can be found in Table 1. The estimated numbers of new HIV infections per year among adults for each country are also included in Table 1. Figures 2, 3 and 4 show the contributions of the six measures of HIV incidence stratified by couple status and source of infection. Over the course of a year of observation, stable concordant HIV-negative couples (that is SCNCs) contribute on average 29.4% of the total HIV population-level incidence across these countries in SSA (Figures 2 and 3). Most new HIV infections among these couples are due to one partner acquiring the infection from a source external to the couple (SCNC_*ext*×1_), with an average of 22.5% (range: 11.1%–39.8%) across all countries (Figure 3A). The average contribution of both partners acquiring the infection from an external source (SCNC_*ext*×2_), within the same year, is less than 1% (range: 0.004%–0.4%) across the countries (Figure 3B). The contribution attributed to the index partner transmitting the infection to the uninfected partner shortly after HIV acquisition from an external source (SCNC_*ext*+*int*_), that is within the same year of observation, is on average 6.8% (range: 3.6%–11.6%) across countries (Figure 3C).

New HIV infections occurring among SDCs identified as sero-discordant at the onset of the year of observation contribute on average 30.4% of the annual total HIV population-level incidence across the countries (Figure 2). Most of these HIV sero-conversions are due to acquiring the infection from the HIV sero-positive partner in the couple (SDC_*int*_; Figure 4A) with an average contribution of 29.7% (range: 9.1%–47.9%). HIV acquisitions from sources external to the couple among SDCs (SDC_*ext*_) contribute minimally to total HIV incidence with an average of 0.7% (range: 0.1%–3.1%) across these countries (Figure 4B). A substantial proportion of HIV incidence occurs among individuals not in SCs (NSC), with a mean of 40.2% (range: 23.7%–64.6%) across countries (Figures 2 and 4C).

**Figure 5:**
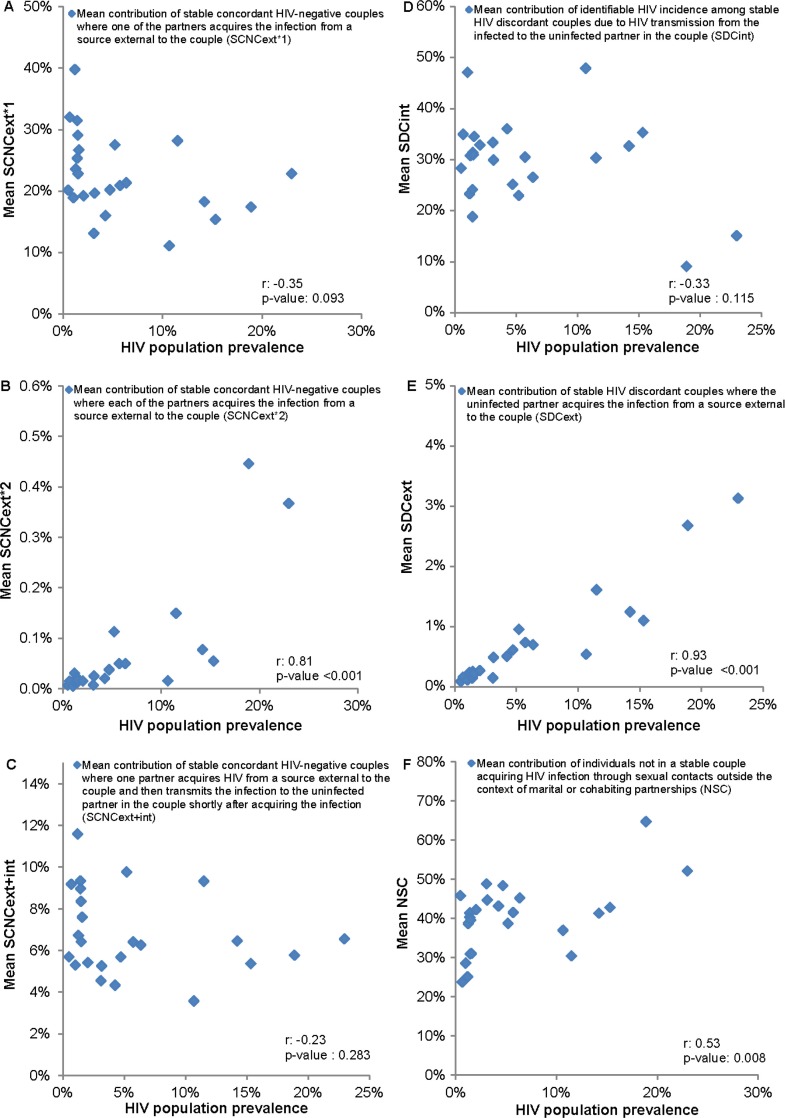
Correlation with HIV prevalence of the mean contribution of: (A) stable concordant HIV-negative couples where one partner acquires the infection from a source external to the couple (SCNC_*ext*×1_), (B) stable concordant HIV-negative couples where each of the partners acquire the infection from a source external to the couple (SCNC_*ext*×2_), (C) stable concordant HIV-negative couples where one partner acquires the infection from a source external to the couple and then transmits it to the uninfected partner in the couple (SCNC_*ext*+*int*_), (D) identifiable HIV incidence among stable HIV discordant couples due to HIV transmission from the infected to the uninfected partner in the couple (SDC_*int*_), (E) HIV incidence among stable HIV discordant couples due to acquiring the infection from a source external to the couple (SDC_*ext*_) and (F) HIV incidence among individuals not in a stable couple (NSC). Values for the Pearson correlation coefficients (r) and their associated *p*-values are incorporated. The analysis discounts the uncertainty in these measures (arising from uncertainty analyses).

Our findings suggest a strong dependence on HIV prevalence for SCNC_*ext*×2_ (Pearson correlation coefficient (*r*): 0.81, *p*<0.001, Figure 5B) and SDC_*ext*_ (*r*: 0.93, *p*<0.001, Figure 5E), where higher contributions are observed in high HIV prevalence countries. NSC is moderately correlated with HIV prevalence (*r*: 0.53, *p*-value: 0.008, Figure 5F). Meanwhile, there is no evident correlation with HIV prevalence for SCNC_*ext*×1_ (*r*: −0.35, *p*-value: 0.093, Figure 5A), SCNC_*ext*+*int*_ (*r*: −0.23, *p*-value: 0.283, Figure 5C) and SDC_*int*_ (*r*: −0.33, *p*-value: 0.115, Figure 5D). The 95% CIs around our estimates for the contributions, generated using the uncertainty analyses, confirmed our finding of roughly equal contribution of SCNCs, SDCs and individuals not in SCs to total HIV incidence in the population across SSA (Figures 3 and 4).

## Discussion

In this article, we complement our earlier work [13–15] by conducting a comprehensive quantitative mapping of the contributions of all sources of HIV exposure to total HIV incidence in the population stratified by couple status. For completeness, we also include updates to few measures reported earlier [13, 14] by analyzing more recent, or first-time available, DHS data for a number of countries and further improving on the precision of some estimates using a more complex mathematical model. Our findings show that close to two-thirds of total HIV incidence every year in SSA occur within the context of marriage or cohabitation, but only half of these infections are actually attributed to HIV transmissions from the infected to the uninfected partner within a couple. The rest of the infections are acquired through extra-partner sexual encounters to the couple. HIV incidence in SSA appears to be roughly equally distributed among concordant HIV-negative couples, HIV sero-discordant couples and individuals not in SCs, with no dominant mode of exposure through which individuals acquire HIV (Figure 2).

Among concordant HIV-negative couples, most HIV incidence is due to one of the partners in the couple acquiring HIV from an external source, while the incidence arising from external HIV acquisitions by both partners within the same year is minimal. HIV transmission to the uninfected partner shortly after the external acquisition of HIV by the other partner in the SCNC, is rather limited at less than 10% of HIV incidence. Meanwhile, HIV sero-conversions among SDCs due to the transmission of the infection from the HIV sero-positive to the HIV sero-negative partner in the couple contribute the majority of HIV incidence among SDCs.

These findings are in close agreement with those measured in the Rakai Study cohort where the contributions of SCNCs, SDCs and individuals not in SCs were 38.5%, 24.0% and 37.6%, respectively (after excluding partnerships with incomplete HIV sero-status information) [33]. Our findings are also in agreement with a recent modeling study that has assessed the incidence contributions among SCs [34]. This study used a mechanistic model that tracks HIV incidence in the population in 18 countries in SSA, as opposed to our programming-oriented approach that is based on a functional definition of incidence among couples in a framework of repeated cross-sectional surveys (Figure 1). Though there are technical differences in the classification of couples’ status and the modes of exposure between the approach of Bellan et al. [34] and that of our study, the findings of both studies converge on the conclusion that HIV incidence in the population is distributed roughly in equal proportions among external infections to couples, within couples and among persons not in couples.

Our updated estimates for HIV incidence among SDCs reaffirm our earlier published work where HIV transmissions from the infected to the uninfected partner in an SDC contributed about a third of new HIV infections arising in the population [13], and the vast majority of HIV incidence arising among SDCs [14]. Indeed, our updated findings for the risk of acquiring the infection externally among SDCs confirm our earlier results as upper-bound estimates for this risk [14].

While SCNCs, SDCs and individuals not in SCs appear to contribute equally to total HIV incidence in the population across SSA, heterogeneity can still be observed across countries (Figure S1 in the Supplementary file). One determinant of the observed differences is the variability in HIV prevalence across Africa (Table 1). This is manifested in the strong association of SCNC_*ext*×2_ (Figures 3B and 5B) and SDC_*ext*_ (Figures 4B and 5E) with HIV population prevalence. Such results are expected as both measures depend proportionally on *λ*, the likelihood of acquiring the infection from a source external to the couple (mathematical expressions in Figure 1), which is higher in high HIV prevalence countries compared to low HIV prevalence countries. The dependence of *λ* on HIV population incidence rate (that is indirectly on HIV population prevalence) also explains the absence of a correlation with HIV prevalence for SCNC_*ext*×1_ (Figures 1, 3A and 5A) and SCNC_*ext*+*int*_ (Figures 1, 3C and 5C). Meanwhile, the moderate correlation with HIV prevalence of the fraction of the population that are engaged in SCs, which appears to be lower in high HIV prevalence countries, is reflected as a moderate correlation between NSC and HIV prevalence (Figures 1, 4C and 5F).

Other factors could also affect the variability of the contribution measures across SSA. These include the level of condom use and coverage of MC in addition to the rates of engagement in SCs across countries (Supplementary file). Condom use and MC within SDCs reduce HIV transmission within SDCs, therefore leading to lower SDC_*int*_ and implicitly higher contributions from HIV infections acquired through sources external to the couple to explain the total observed HIV incidence (Figures S2A and S2B in the Supplementary file). Conversely, higher levels of engagement in SCs are associated with an increase in the contribution of HIV incidence occurring among SCs (Figure S2C in the Supplementary file); so are the increased prevalence of HIV discordancy and HIV-negative concordancy (Figure 1). The impact of variations in these factors on contribution measures is magnified whenever more than one factor is at play. For example, in Swaziland and Lesotho, the world's largest HIV epidemic centres, the higher levels of reported condom use among SCs (Table 1), the lower rates of engagement in SCs (Table 1) and the low prevalence of discordancy among partnerships affected by HIV [1] have yielded lower SDC_*int*_ contribution (Figure 4A), and lower than expected SCNC_*ext*×1_ (Figure 3A) and SCNC_*ext*+*int*_ (Figure 3C) contributions but higher NSC contribution (Figure 4C). Despite the observed heterogeneity, the overall picture appears to suggest an equal contribution of SCNCs, SDCs and individuals not in SCs to total HIV incidence in the population.

One of the highlights of our study is quantifying the contribution of SCNC_*ext*+*int*_. Although, in principle, these infections can be considered as occurring among SDCs instead of SCNCs, the individuals who are newly infected from a source outside the couple are unlikely to be detected within a year by typical counselling and testing programs. We label this type of HIV incidence as *non-identifiable* HIV incidence among SDCs to distinguish it from that where the infected partner transmits HIV to the uninfected partner among couples that have been already identified as SDCs in a cross-sectional survey before HIV transmission to the partner (that is SDC_*int*_). The latter is labelled as *identifiable* HIV incidence among SDCs and is discussed at length in an earlier publication [13]. Our estimates assume annual cross-sectional surveys, but these may not be feasible to implement in resource-limited areas. A lower frequency of cross-sectional surveys would increase the fraction of non-identifiable HIV incidence among SDCs at the expense of the fraction of identifiable HIV incidence among SDCs, making it harder to implement effective SDCs-targeted interventions.

It is worth mentioning that our estimates for the contribution measures factor in implicitly the role of polygamous partnerships in driving HIV incidence among SCs. The DHS-based samples of SCs include each union between a man and a woman at the time of the cross-sectional survey irrespective of the number of concurrent unions.

Our results show that over one-third of HIV population-level incidence occurs among individuals not in SCs (Figures 2 and 4C). This finding is probably not surprising since close to half of HIV incidence in SSA occurs among youths [35–37]. HIV infections arising among young individuals are probably less likely to have been acquired in the context of spousal partnerships and are more likely to have been acquired by other modes of exposure such as through casual or commercial sex encounters.

Although another third of new HIV infections in the population arises from extra-marital partnerships, determining the context in which these infections are acquired, whether through contact with commercial sex networks, other forms of high risk behaviour, or through casual sex, requires an extended mathematical model that includes multiple risk groups and heterogeneity in risk behaviour. Such an analysis is beyond the scope of this publication whose purpose is to quantify the contribution of the different sources of HIV incidence occurring among SCs, rather than investigating the actual drivers of the HIV epidemic across different settings.

The nature of our results where HIV incidence is distributed over different sources of exposure limits our ability to develop specific HIV policy and programming recommendations for SSA and suggests the need to simultaneously address the different modes of HIV exposure in the population. A treatment as prevention (TasP) approach [38], and the new World Health Organization treatment recommendations of increasing ART coverage to include HIV infected individuals with CD4 count<500 [39], should achieve substantial reductions in HIV transmission across all sources of exposure to HIV infection. However, the specific impact of such interventions on HIV incidence through each source of exposure is not yet known. ART coverage may vary from one sub-population to another due to variable access to voluntary counselling and testing services. Maintaining HIV infected individuals in the treatment cascade may also vary across sub-populations [40]. The impact of the interventions on the onward transmission of HIV in the population is also not yet entirely clear. With the increasing availability of data on ART coverage, more complex mathematical models can be designed to elucidate the impact of different HIV interventions on HIV incidence through each of these modes of exposure.

Our estimates are affected by the representativeness and precision of available data. The recency of the epidemiological and behavioural parameters used in our analyses was determined by the availability of HIV biomarker information in the DHS surveys. However, DHS surveys are increasingly being implemented in SSA and only few surveys of those used in our analysis were older than five years [16]. For some countries, data were only available in small sample sizes resulting in wide CIs around some of the measures. Our estimates could also have been affected by inherent biases in the DHS data such as the variability in response rate to HIV testing [41], and selection bias in restricting our analysis to couples with complete HIV sero-status information. Despite these limitations, DHS surveys are the only standardized nationally representative surveys conducted consistently across the countries of SSA [16].

There are heterogeneities in risk behaviour and not all of the population in reproductive age is necessarily sexually active. Nevertheless, the measures that we used to parameterize our model are averages for the specific sub-populations of interest regardless of the heterogeneities within. Heterogeneities can affect the estimates for a specific sub-group of a sub-population of interest, but they may not affect the average for the whole sub-population. This is especially true because the focus of our study is on the short-term HIV incidence within a year of observation where non-linear effects that are sensitive to variability are not at play. Long-term projections, however, including HIV onward transmission, could potentially be affected by variability in risk behaviour.

Epidemic type or phase can affect the distribution of HIV infection in a population, and potentially our results. Nonetheless, our model uses empirical cross-sectional data of HIV distribution and discordancy for a specific year, and epidemic type and phase implicitly affects and drives these data. Therefore effects due to epidemic type or phase should be implicitly accounted for in our calculations.

We parameterized our model using the HIV transmission probability per coital act (*p*) as measured in the Rakai Study [22] and the Partners in Prevention Study [23, 24] which, to date, are the best available empirical evidence for this measure. Yet, different biological or behavioural factors may affect *p* across settings. We have already accounted in our calculations for the coverage of MC and the uptake of condom use among SCs in each country (Table 1). Still, other factors may affect *p* but are difficult to adjust for due to data limitations such as the presence of sexually transmitted infections other than HIV [42, 43], other co-infections that increase HIV viral load [44, 45], viral factors [46–48], differences in viral sub-types that may lead to longer period of elevated viral concentration in the early stages of HIV infection [48] and host genetics and immunology [46]. Our recent analysis of the risk of HIV transmission within SDCs in SSA suggests substantial variability across countries [15]. Incorporation of such variability can affect the reported estimates by increasing the contribution of within-couple transmission, wherever the risk of HIV transmission is higher, and reducing the within couple HIV transmission, wherever the risk of HIV transmission is lower. Similarly, if the country-specific HIV population incidence rates were actually lower than those estimated by the SPECTRUM model or derived using the DHS HIV prevalence, this would increase the contribution of within couple HIV transmission at the expense of other contributions.

We also parameterized our model using *p* during acute infection as derived by Pinkerton using the Rakai cohort data [22, 25]. This value is similar to those reported by Hollingsworth et al. [49], Powers et al. [50] and Boily et al. [51]. Given the small contribution of SCNC_*ext*+*int*_ (Figures 2 and 3C), it is not likely that the uncertainty in *p* during acute infection [52] will affect our findings.

In the absence of country-specific empirical measures, we assumed that the risk of HIV acquisition among individuals not in SCs is equal to the HIV population-level incidence rate. This assumption is reasonable on balance of our knowledge of HIV epidemiology among persons in SCs and not in SCs, and the fact that individuals not in SCs constitute a large fraction of the population in reproductive age, nearly half of it, across countries. Empirical data, such as those of the Rakai Study [33], as well as HIV incidence age-distribution patterns in SSA that are skewed towards young age [53], support also the plausibility of this assumption.

We structured our model to derive the probability of acquiring HIV from sources external to the couple (*λ*) and hence, increase the precision of our contribution measures. Our estimates for the average *λ* as calculated using 10,000 runs of model fits were reasonable in terms of their scale, since, as expected, they were close yet smaller than the HIV population-level incidence rate estimated through SPECTRUM or derived from DHS.

The structure of our model does not consider variations in partnership duration among SCs. However, the long durations of stable partnerships [15], and the large rates of engagement in SCs across SSA (Table 1), suggest that partnership formation and dissolution within the course of a year of observation would not be substantial enough to affect our findings.

Finally, we have relied on a volume of data sources derived using different methodologies to draw a comprehensive assessment for the contribution of SCs to the HIV epidemic across the African continent. This may have potentially led to inconsistencies that could impact our predictions. Though the above mentioned limitations may have affected the precision of our quantitative results, our uncertainty analyses suggest that they are not likely to affect our findings that no single mode of exposure among SCs contributes the majority of HIV incidence that is occurring in SSA.

In conclusion, we presented a comprehensive mapping of the contribution of SCs to HIV incidence in SSA. Our estimates were based on a mathematical model parameterized by state-of-the-art empirical and nationally-representative population-based data. We conclude that no single mode of exposure among SCs contributes to the majority of HIV incidence. Accordingly, a multi-focus HIV prevention strategy that optimizes the use of available prevention interventions by targeting the different modes of exposure to HIV in the population, each according to its contribution weight, is needed to address the HIV epidemic in SSA. Further translational research and intervention impact assessments are needed to delineate more specific recommendations for HIV policy and programming.

## References

[1] Chemaitelly H, Cremin I, Shelton JD, Hallett TB, Abu-Raddad LJ (2012). Distinct HIV discordancy patterns by epidemic size in stable sexual partnerships in sub-Saharan Africa. Sex Transm Infect.

[2] Guthrie BL, De Bruyn G, Farquhar C (2007). HIV-1-discordant couples in sub-Saharan Africa: explanations and implications for high rates of discordancy. Curr HIV Res.

[3] Lingappa JR, Lambdin B, Bukusi EA, Ngure K, Kavuma L, Inambao M (2008). Regional differences in prevalence of HIV-1 discordance in Africa and enrollment of HIV-1 discordant couples into an HIV-1 prevention trial. PLoS One.

[4] Bunnell R, Opio A, Musinguzi J, Kirungi W, Ekwaru P, Mishra V (2008). HIV transmission risk behavior among HIV-infected adults in Uganda: results of a nationally representative survey. AIDS.

[5] Baryarama F, Bunnell R, McFarland W, Hudes ES, Neilands TB, Ransom RL (2007). Estimating HIV incidence in voluntary counseling and testing clients in Uganda (1992–2003). J Acquir Immune Defic Syndr.

[6] Abdool Karim Q, Abdool Karim SS, Frohlich JA, Grobler AC, Baxter C, Mansoor LE (2010). Effectiveness and safety of tenofovir gel, an antiretroviral microbicide, for the prevention of HIV infection in women. Science.

[7] Cohen MS, Chen YQ, McCauley M, Gamble T, Hosseinipour MC, Kumarasamy N (2011). Prevention of HIV-1 infection with early antiretroviral therapy. N Engl J Med.

[8] Thigpen MC, Kebaabetswe PM, Paxton LA, Smith DK, Rose CE, Segolodi TM (2012). Antiretroviral preexposure prophylaxis for heterosexual HIV transmission in Botswana. N Engl J Med.

[9] Baeten JM, Donnell D, Ndase P, Mugo NR, Campbell JD, Wangisi J (2012). Antiretroviral prophylaxis for HIV prevention in heterosexual men and women. N Engl J Med.

[10] UNAIDS, WHO, UNICEF (2011). Global HIV/AIDS response: epidemic update and health sector progress towards universal access.

[11] UNAIDS (2012). UNAIDS world AIDS day report.

[12] World Health Organization (2012). Guidance on couples HIV testing and counselling including antiretroviral therapy for treatment and prevention in serodiscordant couples.

[13] Chemaitelly H, Shelton JD, Hallett TB, Abu-Raddad LJ (2013). Only a fraction of new HIV infections occur within identifiable stable discordant couples in sub-Saharan Africa. AIDS.

[14] Chemaitelly H, Abu-Raddad LJ (2013). External infections contribute minimally to HIV incidence among HIV sero-discordant couples in sub-Saharan Africa. Sex Transm Infect.

[15] Chemaitelly H, Awad S, Abu-Raddad LJ (2014). The risk of HIV transmission within HIV-1 sero-discordant couples appears to vary across sub-Saharan Africa. Epidemics.

[16] MEASURE DHS Demographic and health surveys [Internet]. http://www.measuredhs.com/data/available-datasets.cfm.

[17] United Nations Department of Economic and Social Affairs, Population Division, Population Estimates and Projections Section World population prospects: the 2010 revision population database. http://esa.un.org/unpd/wpp/unpp/panel_population.htm.

[18] Rutstein S, Rojas G (2006). Guide to DHS statistics.

[19] Demographic and Health Surveys Dataset FAQs: dataset indicators [Internet]. http://www.measuredhs.com/accesssurveys/dataset_faqs.cfm.

[20] UNAIDS HIV estimates with uncertainty bounds 1990–2011. http://www.unaids.org/en/resources/campaigns/20121120_globalreport2012/globalreport/.

[21] Nelson KE, Williams CM (2007). Infectious disease epidemiology: theory and practice.

[22] Wawer MJ, Gray RH, Sewankambo NK, Serwadda D, Li X, Laeyendecker O (2005). Rates of HIV-1 transmission per coital act, by stage of HIV-1 infection, in Rakai, Uganda. J Infect Dis.

[23] Celum C, Wald A, Lingappa JR, Magaret AS, Wang RS, Mugo N (2010). Acyclovir and transmission of HIV-1 from persons infected with HIV-1 and HSV-2. N Engl J Med.

[24] Hughes JP, Baeten JM, Lingappa JR, Magaret AS, Wald A, de Bruyn G (2012). Determinants of Per-Coital-Act HIV-1 infectivity among African HIV-1-serodiscordant couples. J Infect Dis.

[25] Pinkerton SD (2008). Probability of HIV transmission during acute infection in Rakai, Uganda. AIDS Behav.

[26] Hughes JP (2010). Personal communication.

[27] Eyawo O, de Walque D, Ford N, Gakii G, Lester RT, Mills EJ (2010). HIV status in discordant couples in sub-Saharan Africa: a systematic review and meta-analysis. Lancet Infect Dis.

[28] Weller S, Davis K (2001). Condom effectiveness in reducing heterosexual HIV transmission. Cochrane Database Syst Rev.

[29] Weiss HA, Halperin D, Bailey RC, Hayes RJ, Schmid G, Hankins CA (2008). Male circumcision for HIV prevention: from evidence to action?. AIDS.

[30] Auvert B, Taljaard D, Lagarde E, Sobngwi-Tambekou J, Sitta R, Puren A (2005). Randomized, controlled intervention trial of male circumcision for reduction of HIV infection risk: the ANRS 1265 Trial. PLoS Med.

[31] Bailey RC, Moses S, Parker CB, Agot K, Maclean I, Krieger JN (2007). Male circumcision for HIV prevention in young men in Kisumu, Kenya: a randomised controlled trial. Lancet.

[32] Gray RH, Kigozi G, Serwadda D, Makumbi F, Watya S, Nalugoda F (2007). Male circumcision for HIV prevention in men in Rakai, Uganda: a randomised trial. Lancet.

[33] Gray R, Ssempiija V, Shelton J, Serwadda D, Nalugoda F, Kagaayi J (2011). The contribution of HIV-discordant relationships to new HIV infections in Rakai, Uganda. AIDS.

[34] Bellan SE, Fiorella KJ, Melesse DY, Getz WM, Williams BG, Dushoff J (2013). Extra-couple HIV transmission in sub-Saharan Africa: a mathematical modelling study of survey data. Lancet.

[35] Hallett TB, Zaba B, Todd J, Lopman B, Mwita W, Biraro S (2008). Estimating incidence from prevalence in generalised HIV epidemics: methods and validation. PLoS Med.

[36] Gouws E, Williams BG, Sheppard HW, Enge B, Karim SA (2002). High incidence of HIV-1 in South Africa using a standardized algorithm for recent HIV seroconversion. J Acquir Immune Defic Syndr.

[37] Monasch R, Mahy M (2006). Young people: the centre of the HIV epidemic. World Health Organ Tech Rep Ser.

[38] Granich RM, Gilks CF, Dye C, De Cock KM, Williams BG (2009). Universal voluntary HIV testing with immediate antiretroviral therapy as a strategy for elimination of HIV transmission: a mathematical model. Lancet.

[39] World Health Organization (2013). Consolidated guidelines on the use of antiretroviral drugs for treating and preventing HIV infection.

[40] Kilmarx PH, Mutasa-Apollo T (2013). Patching a leaky pipe: the cascade of HIV care. Curr Opin HIV AIDS.

[41] Mishra V, Barrere B, Hong R, Khan S (2008). Evaluation of bias in HIV seroprevalence estimates from national household surveys. Sex Transm Infect.

[42] Abu-Raddad LJ, Magaret AS, Celum C, Wald A, Longini IM, Self SG (2008). Genital herpes has played a more important role than any other sexually transmitted infection in driving HIV prevalence in Africa. PLoS One.

[43] Korenromp EL, de Vlass SJ, Nagelkerke NJ, Habbema JD (2001). Estimating the magnitude of STD cofactor effects on HIV transmission: how well can it be done?. Sex Transm Dis.

[44] Abu-Raddad LJ, Patnaik P, Kublin JG (2006). Dual infection with HIV and malaria fuels the spread of both diseases in sub-Saharan Africa. Science.

[45] Abu-Raddad LJ, Barnabas RV, Janes H, Weiss HA, Kublin JG, Longini IM (2013). Have the explosive HIV epidemics in sub-Saharan Africa been driven by higher community viral load?. AIDS.

[46] Kaul R, Cohen CR, Chege D, Yi TJ, Tharao W, McKinnon LR (2011). Biological factors that may contribute to regional and racial disparities in HIV prevalence. Am J Reprod Immunol.

[47] Novitsky V, Woldegabriel E, Kebaabetswe L, Rossenkhan R, Mlotshwa B, Bonney C (2009). Viral load and CD4+ T-cell dynamics in primary HIV-1 subtype C infection. J Acquir Immune Defic Syndr.

[48] Novitsky V, Ndung'u T, Wang R, Bussmann H, Chonco F, Makhema J (2011). Extended high viremics: a substantial fraction of individuals maintain high plasma viral RNA levels after acute HIV-1 subtype C infection. AIDS.

[49] Hollingsworth TD, Anderson RM, Fraser C (2008). HIV-1 transmission, by stage of infection. J Infect Dis.

[50] Powers KA, Ghani AC, Miller WC, Hoffman IF, Pettifor AE, Kamanga G (2011). The role of acute and early HIV infection in the spread of HIV and implications for transmission prevention strategies in Lilongwe, Malawi: a modelling study. Lancet.

[51] Boily MC, Baggaley RF, Wang L, Masse B, White RG, Hayes RJ (2009). Heterosexual risk of HIV-1 infection per sexual act: systematic review and meta-analysis of observational studies. Lancet Infect Dis.

[52] Cohen MS, Dye C, Fraser C, Miller WC, Powers KA, Williams BG (2012). HIV treatment as prevention: debate and commentary – will early infection compromise treatment-as-prevention strategies?. PLoS Med.

[53] Hallett TB, Stover J, Mishra V, Ghys PD, Gregson S, Boerma T (2010). Estimates of HIV incidence from household-based prevalence surveys. AIDS.

